# Effects of Music and Aromatherapy on Blood Pressure and Heart Rate Among Endodontic Patients: A Randomized Clinical Trial

**DOI:** 10.1002/cre2.70156

**Published:** 2025-06-11

**Authors:** Seyed Amir Mousavi, Fateme Nasiri, Melika Sadat Araghbidi Kashani, Ali Ghazalgoo, Pedram Iranmanesh, Soheil Shahbazi

**Affiliations:** ^1^ Department of Endodontics, School of Dentistry Meharry Medical College Nashville Tennessee USA; ^2^ Department of Endodontics, Dental Research Center, School of Dentistry, Dental Research Institute Isfahan University of Medical Sciences Isfahan Iran; ^3^ Dental Research Center, Research Institute of Dental Sciences Shahid Beheshti University of Medical Sciences Tehran Iran

**Keywords:** anxiety, aromatherapy, music, root canal therapy

## Abstract

**Objectives:**

Music and aromatherapy are recognized as noninvasive methods for promoting relaxation. Endodontic treatment is often perceived as a stressful procedure in dental settings. This study aimed to assess the effects of these interventions on blood pressure and heart rate among patients undergoing root canal therapy.

**Material and Methods:**

In this single‐blind, parallel‐group, randomized clinical trial, 72 adult patients diagnosed with irreversible pulpitis and normal periapical conditions were referred to a dental school for endodontic treatment. Participants were randomly assigned to one of four groups: (1) control group, (2) music group (participants listened to tracks of Stefano Crespan Shantam, tuned to 432 Hz), (3) aromatherapy group (participants were exposed to five drops of lavender oil via a humidifier), and (4) combined music and aromatherapy group. Blood pressure and heart rate were measured before and after the intervention. Data were analyzed using SPSS software and one‐way ANOVA.

**Results:**

Although systolic and diastolic blood pressure, as well as heart rate, decreased in the intervention groups, there were no statistically significant differences compared to the control group (*p* > 0.05).

**Conclusions:**

Music and aromatherapy did not produce statistically significant reductions in blood pressure and heart rate among endodontic patients. Alternative methods should be explored to manage patient anxiety better.

**Trial Registration:**

The present trial was registered in the Registry of Clinical Trials (IRCT20191228045910N1), available at https://www.irct.ir/trial/46195.

## 1 Background

Patients undergoing endodontic treatment may experience varying degrees of anxiety due to both procedural and non‐procedural factors (Olivieri et al. 2021). Dental anxiety is a well‐documented barrier to care, often leading to avoidance of treatment, as well as increased intraoperative and postoperative pain (Murillo‐Benítez et al. 2020; Vitali et al. 2024). Elevated anxiety levels can also result in diagnostic inaccuracies and the development of suboptimal treatment plans (Eli 1993). Conversely, effective management of patient anxiety facilitates regular dental attendance, improves oral hygiene behaviors, and enhances overall quality of life (Walsh 2007). To this end, a variety of nonpharmacological strategies—such as music and aromatherapy—have been proposed to alleviate anxiety in dental settings (Di Nasso et al. 2016; Malamed 2017; Morarend et al. 2011; Hao et al. 2023; Qazi et al. 2024).

Physiological markers like blood pressure and heart rate are well‐established indicators of anxiety, especially in clinical dental settings (Özmen and Taşdemir 2024). Anxiety activates the sympathetic nervous system, producing measurable cardiovascular responses that serve as valid proxies for emotional arousal during procedures such as endodontic treatment (Özmen and Taşdemir 2024). Although conditions like white coat hypertension and posttraumatic stress disorder (PTSD) may also affect these metrics, their underlying triggers differ. White coat hypertension involves transient blood pressure spikes in response to clinical environments, typically without psychological distress. Procedural anxiety, by contrast, reflects anticipatory fear specific to the treatment itself and is marked by short‐term physiological arousal (Carter et al. 2015). PTSD is a chronic disorder involving persistent hyperarousal and trauma‐related reactivity, unrelated to routine clinical procedures (Astudillo et al. 2024). Although autonomic responses may overlap, these conditions differ significantly in etiology, duration, and clinical relevance to dental care.

Music therapy, in particular, has been widely adopted in medical contexts to address patients' physiological, psychological, and emotional needs (Dileo and Bradt 2005). Numerous studies over the past two decades have demonstrated its efficacy in reducing anxiety levels across diverse patient populations (Dileo and Bradt 2005; Mok and Wong 2003; Hamel 2001; Olszewska and Zarow 2003). More recently, artificial intelligence technologies have also been explored for predicting dental anxiety, further reflecting the growing interest in this area (Ogwo et al. 2024). Additionally, auditory stimuli—such as the sounds associated with dental equipment—have been identified as significant contributors to dental anxiety during endodontic procedures (Khoo et al. 2022; Ghaffar et al. 2024). Evidence supports the role of music in mitigating such stressors, with reported benefits, including decreased surgical stress, enhanced relaxation, and reductions in blood pressure, heart rate, and respiratory rate during procedures performed under local anesthesia (Jones 2013; Guzzetta 1991; Kim et al. 2011).

Aromatherapy has been proposed by some clinicians as an effective method for reducing pain, alleviating anxiety, and promoting healing. However, the underlying mechanisms responsible for these purported benefits remain inadequately understood. One prevailing theory posits that aromatic compounds may elicit favorable pharmacological and physiological responses (Dobetsberger and Buchbauer 2011). Although the medical literature contains substantial evidence supporting the general benefits of aromatherapy (Dobetsberger and Buchbauer 2011; Ndao et al. 2012; Lehrner et al. 2005), its specific impact on dental anxiety is less well established. Various substances have been used in aromatherapy, with lavender being among the most frequently studied; however, findings regarding its efficacy remain inconsistent across studies (Janthasila and Keeratisiroj 2023; Pradopo et al. 2017; Packyanathan et al. 2019; James et al. 2021; Jaafarzadeh et al. 2013; Waheed et al. 2022; Maybodi et al. 2018).

A systematic review suggests that aromatherapy may be more effective than control conditions in reducing dental anxiety and yields results comparable to those of music‐based interventions. Nevertheless, its use in dental practice—particularly within the context of endodontics—has not been thoroughly investigated. Moreover, much of the existing evidence is drawn from studies involving procedures other than root canal therapy and may be affected by methodological bias (Cai et al. 2021). Therefore, the present study aims to evaluate the potential effects of music and aromatherapy on blood pressure and heart rate among patients undergoing endodontic treatment.

## 2 Methods

### 2.1 Registration

The present trial was registered in the Registry of Clinical Trials (IRCT20191228045910N1), available at https://www.irct.ir/trial/46195. Ethical approval was obtained from the local institutional review board (IR.MUI.RESEARCH.REC.1398.526), and written informed consent was obtained from all participants before enrollment. The study adheres to the CONSORT 2010 reporting guidelines (File S1).

### 2.2 Sample Recruitment

Participants were recruited from adult patients (aged ≥ 18 years) referred to the dental school clinic, diagnosed with irreversible pulpitis, and who presented with normal periapical conditions. The diagnosis of irreversible pulpitis was confirmed using a cold test with ENDO‐FROST spray (Coltène‐Whaledent, Langenau, Germany), and normal periapical status was determined based on the absence of sensitivity to percussion or palpation, along with a periapical index score of 1. Inclusion criteria required that participants be non‐smokers with intact olfactory and auditory functions and without any known allergies or psychiatric disorders. Exclusion criteria included the presence of upper respiratory infections or allergies, use of analgesics or sedatives before the procedure, requirement of more than one cartridge of local anesthetic, occurrence of intraoperative emergencies, or procedures lasting longer than 1 h.

### 2.3 Study Design

This single‐blind, parallel‐group, superiority randomized clinical trial included 72 participants, who were equally and randomly assigned to one of four groups using the permuted block method: (1) control group, (2) music group, (3) aromatherapy group, and (4) combined music and aromatherapy group. The sample size calculation formula used was as follows: *n* = (*z*_1_ + *z*_2_)^2^ × (*S*_1_^2^ + *S*_2_^2^)/*d* ^2^, where *z*_1_ represents *z*(1 − *α*/2) and *z*_2_ represents *z*(1 − *β*). Given *α* = 0.05 and *β* = 0.2 (i.e., 80% power), *z*_1_ = 1.96 and *z*_2_ = 0.84. The variance of the dependent variable in previous research was 0.738 for *S*_1_ and 0.634 for *S*_2_ (Pradopo et al. 2017; Jaafarzadeh et al. 2013). With an assumed effect size of ≈0.45 (*d* ^2^ = 0.2), the sample size was determined to be 72, resulting in 18 individuals in each of the four groups (Levy and Lemeshow 2013). Allocation concealment was maintained using sequentially numbered, sealed envelopes distributed by an investigator (F.N.). Recruitment and randomization were overseen by a senior investigator (A.G.).

### 2.4 Intervention

Endodontic procedures were performed by two experienced endodontists (A.G. and S.A.M.). After administering 1.8 mL of 2% lidocaine hydrochloride with 1:80,000 epinephrine (DarouPakhsh, Tehran, Iran), teeth were isolated using a rubber dam (Sanctuary, Perak, Malaysia). Access cavities were prepared, and coronal flaring was performed using the SX ProTaper Universal rotary file (Dentsply Sirona, Ballaigues, Switzerland). Working length was determined using an apex locator (Morita, Tokyo, Japan). Root canals were instrumented using S1, S2, F1, and F2 ProTaper Universal rotary files via the crown‐down technique. Irrigation was carried out with 2.5% sodium hypochlorite delivered through a 27‐gauge side‐vented needle (Ultradent, South Jordan, UT, USA). Obturation was completed via lateral compaction using the AH26 sealer (Dentsply, Tulsa Dental, Tulsa, OK, USA) and gutta‐percha (Meta Biomed, Chungcheongbuk‐do, Korea).

In the music group, patients selected their preferred track from five instrumental pieces by Stefano Crespan Shantam, ranging from 7 to 27 min in duration (Di Nasso et al. 2016). Music was played via office speakers at a volume of approximately 60 dB, and the control for aromatherapy (distilled water) was used to maintain experimental consistency. The music was adjusted to 432 Hz, which is considered the standard tuning pitch (Di Nasso et al. 2016). For the aromatherapy group, five drops of lavender essential oil (Keshtosanat Kazeroon) were placed in a humidifier (Shamim, Tehran University) to disperse the aroma in a 20 m² treatment room. The combined group received both interventions simultaneously, whereas the control group was not exposed to music or aroma. A flow diagram of participant allocation and study procedures is provided in File S2.

### 2.5 Outcomes and Statistics

Systolic and diastolic blood pressure, as well as heart rate, were recorded at two time points: before the administration of local anesthesia and after placement of the temporary filling. These measurements were obtained using an electronic sphygmomanometer (Omron Hem‐7111, Omron Healthcare Co. Ltd., Kyoto, Japan) by a blinded examiner (F.N.). Statistical analysis was conducted using SPSS version 24 (IBM Corp., Armonk, NY, USA), using *χ* ^2^ tests and one‐way ANOVA to compare outcomes across groups.

## 3 Results

There were no statistically significant differences in demographic characteristics among the groups at baseline (Table 1). Similarly, no significant differences were observed in systolic blood pressure, diastolic blood pressure, or heart rate across the groups before treatment (*p* > 0.05, Table 2).

**Table 1:** Baseline demographics of the groups.

Groups	Mean age (SD)	Male/female
Control	36.56 (18.35)	6/12
Music	37.5 (15.77)	5/13
Aromatherapy	38.39 (18.11)	4/14
Combined music and aromatherapy	39.44 (16.17)	10/8
*p* value	0.087^a^	0.063^b^

^a^ One‐way ANOVA.

^b^ *χ*².

**Table 2:** Blood pressure and heart rate of groups before and after the treatment.

Groups	SBP (mmHg)				DBP (mmHg)				Heart rate (bpm)			
	Before		After		Before		After		Before		After	
	Mean (SD)	95% CI	Mean (SD)	95% CI	Mean (SD)	95% CI	Mean (SD)	95% CI	Mean (SD)	95% CI	Mean (SD)	95% CI
Control	114.8 (15.8)	106.9–122.6	114.2 (19.1)	104.7–123.6	70.7 (11.7)	64.8–76.5	69.3 (11.1)	63.7–74.8	84.8 (9.7)	79.9–89.6	80.2 (11.6)	74.5–86.0
Music	116.5 (9.4)	111.8–121.1	115.7 (11.8)	109.8–121.5	73.6 (9.4)	68.9–78.2	72.8 (9.4)	68.1–77.4	84.2 (11.1)	78.6–89.7	79.2 (10.0)	74.2–84.2
Aromatherapy	109.0 (20.3)	98.9–119.0	108.3 (19.1)	98.8–117.7	70.5 (9.4)	65.8–75.1	69.1 (9.4)	64.4–73.7	83.0 (11.6)	77.2–88.8	78.5 (8.9)	74.0–83.0
Combined music and aromatherapy	114.8 (12.4)	108.6–120.9	119.8 (16.4)	111.6–127.9	73.8 (8.7)	69.4–78.1	71.4 (10.7)	66.0–76.7	82.0 (13.7)	78.2–88.8	77.2 (12.0)	71.2–83.2
*p* value^a^	0.059		0.066		0.089		0.087		0.079		0.061	

Abbreviations: CI, confidence interval; DBP, diastolic blood pressure; SBP, systolic blood pressure; SD, standard deviation.

^a^ One‐way ANOVA.

**Figure 1:**
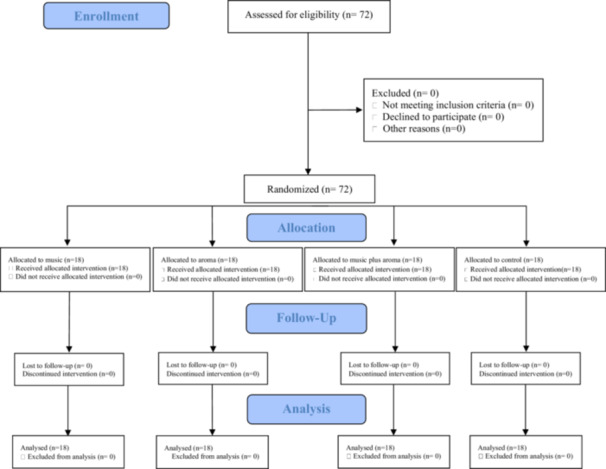
CONSORT flow diagram.

All 72 participants completed the study and were included in the final statistical analysis (Figure 1). Although posttreatment measurements indicated a reduction in systolic and diastolic blood pressure as well as heart rate in all three intervention groups, these changes were not statistically significant when compared to the control group (*p* > 0.05, Table 2). No adverse effects were reported by any of the participants at the end of the treatment session.

## 4 Discussion

Music and aromatherapy have been proposed as nonpharmacological interventions to reduce patient anxiety in both medical and dental contexts. Music can serve as a distraction and a source of comfort while minimizing environmental noise within the clinical setting. Aromatherapy provides a noninvasive approach to anxiety management by masking clinical odors and fostering a more pleasant sensory experience, potentially forming positive associations for patients (Armfield et al. 2007). This randomized clinical trial evaluated the effects of music and aromatherapy on blood pressure and heart rate in patients undergoing endodontic treatment. The findings indicated that neither intervention produced a statistically significant reduction in these physiological parameters.

Numerous studies have investigated the impact of music and aromatherapy on dental anxiety (Table 3). The majority of research focused on music therapy has demonstrated its efficacy in reducing anxiety, particularly among pediatric patients undergoing preventive or minimally invasive procedures (Janthasila and Keeratisiroj 2023; Pradopo et al. 2017; Packyanathan et al. 2019; James et al. 2021; Jaafarzadeh et al. 2013; Waheed et al. 2022; Maybodi et al. 2018). However, patients often report that the anxiety associated with nonsurgical root canal treatment is comparable to that experienced during oral surgical procedures (Khan et al. 2016). Therefore, assessing the effectiveness of these interventions in the context of more invasive treatments remains an important area of investigation.

**Table 3:** Characteristics of studies regarding the application of music or aromatherapy in the dental setting.

Author (year)	Ages (year)	Sample size for each group	Intervention place	Odor (extract/drops)	Music (type/playing parameters)	Dental treatment	Parameters	Conclusion
Janthasila and Keeratisiroj 2023	10–12	33	Service room	Lavender	Thai pop/60 dB	Sealant services	Blood pressure, heart rate, oxygen saturation	Reduced anxiety and fear
Waheed et al. 2022	20–26	30	Treatment room	Strawberry	NR/NR	Restorative	Pain intensity scale, pain severity scale	Music reduced anxiety and pain
James et al. 2021	6–8	50	Treatment room	Orange	NR/NR	Restorative	Pulse rate, respiratory rate, oxygen saturation, Venham's picture test	Reduced anxiety
Packyanathan et al. 2019	17–64	25	Before and during extraction	NA	Classical music/NR	Extraction	Systolic pressure, diastolic pressure, heart rate	Reduced anxiety
Maybodi et al. 2018	Adults	30	Treatment room	Lavender/10 drops	Relaxing music/NR	Crown lengthening	Spielberger's questionnaire, Blood pressure, heart rate	Aroma reduced anxiety
Pradopo et al. 2017	5–7	20	During treatment	Pandan leaves	Classic/NR	Fissure sealant	Blood pressure, pulse rate	Reduced anxiety
Jaafarzadeh et al. 2013	6–9	15	Operation room	Orange	NA	Fissure sealant	Salivary cortisol, pulse rate	Reduced anxiety

Abbreviations: NA, not applicable; NR, not reported.

In the present study, music was played through external speakers in the treatment room rather than headphones to maintain open verbal communication between the patient and the clinician. The use of headphones was avoided, as it may potentially increase anxiety by limiting interaction with the practitioner (Bradt and Teague 2018). However, some studies suggest that headphones equipped with noise cancellation or volume control may offer additional benefits, such as minimizing distractions and preserving staff concentration during procedures (Kim et al. 2011; Aitken et al. 2002). According to Nilsson, music interventions are most effective when the music is instrumental, selected by the patient, played at a volume of approximately 60 dB, and has a tempo ranging between 60 and 90 beats per minute—all of which were applied in the current study design (Nilsson 2008). From a musicological perspective, 432 Hz tuning aligns closely with the natural resonant frequencies of the human body (Di Nasso et al. 2016). Several studies have reported that music tuned to this frequency may effectively reduce anxiety and lower cortisol levels in dental patients undergoing various procedures, including extractions, oral surgeries, and root canal treatments (Di Nasso et al. 2016; Menziletoglu et al. 2021; Aravena et al. 2020). Notably, many previous studies did not specify the type or parameters of music used, nor did they consistently report the setting in which the intervention was administered (e.g., waiting room vs. treatment room). Such variations in intervention delivery may contribute to the heterogeneity of findings across studies. Furthermore, incorporating additional sensory modalities—such as visual stimuli via audiovisual resources—has been shown to enhance the calming effect of music and further reduce patient anxiety during endodontic procedures (Craveiro and Caldeira 2020).

Regarding the application of aromatherapy in a dental setting, Alkanan et al. (Alkanan et al. 2023). demonstrated that it significantly reduced anxiety scores during minor to moderately stressful dental procedures and lowered pain perception by approximately 50% compared to control groups. Most available evidence supports its effectiveness in reducing anxiety among pediatric patients undergoing short, simple treatments such as pit and fissure sealant application or restorative procedures (Janthasila and Keeratisiroj 2023; Pradopo et al. 2017; Packyanathan et al. 2019; James et al. 2021; Jaafarzadeh et al. 2013; Waheed et al. 2022; Maybodi et al. 2018). The characteristic clinical odors present in dental environments—such as eugenol, disinfectants, and removed dentin—are known to provoke anxiety, and this effect may be mitigated by masking these odors through aromatherapy (Armfield et al. 2007). It is important to note, however, that existing studies vary widely in terms of the aromatic agents and concentrations used. Additionally, inconsistencies in treatment duration and the frequency of aroma replenishment were not adequately reported in several studies (Table 3). These methodological differences likely contributed to the variability in study outcomes.

Cognitive conditioning and parental influence are recognized as primary contributors to anxiety associated with root canal treatment. This anxiety often manifests through heightened sympathetic nervous system activity and increased peripheral vascular resistance within the dental environment (Carter et al. 2015; Astudillo et al. 2024). As a result, blood pressure and heart rate are frequently used as objective, numerical indicators of patient anxiety in clinical studies (Özmen and Taşdemir 2024; Janthasila and Keeratisiroj 2023; Pradopo et al. 2017; Packyanathan et al. 2019; James et al. 2021; Jaafarzadeh et al. 2013; Maybodi et al. 2018). However, the extent to which these physiological measures accurately reflect anxiety remains a subject of ongoing debate. Dantas et al (Dantas et al. 2017). reported a positive correlation between anxiety levels and blood pressure, whereas Gil‐Abando et al (Gil‐Abando et al. 2022). observed only weak associations between dental anxiety and heart rate. Consequently, some researchers advocate for the use of validated dental anxiety scales as more reliable tools for assessing anxiety. Nevertheless, the current literature has not established a universally accepted “gold standard” for measuring dental anxiety (Chi 2023; Al‐Namankany et al. 2012). The present study focused on evaluating objective and quantifiable physiological indicators—namely, blood pressure and heart rate—as proxies for anxiety. Although such measures can offer valuable insights, they may be limited by individual physiological variability and influenced by factors unrelated to anxiety. Moreover, although behavioral observations of anxiety based on physiological responses are often utilized, they remain subject to interpretive bias. Future research would benefit from integrating standardized behavioral assessments alongside physiological metrics to provide a more comprehensive evaluation of patient anxiety.

Additionally, patients taking analgesic or sedative medications were excluded from this study to minimize confounding factors that could independently alter anxiety levels. Although these individuals are more likely to experience elevated dental anxiety (Halonen et al. 2018), their inclusion could have biased the results and hindered an accurate assessment of the effects of the music and aromatherapy interventions.

### 4.1 Limitations and Future Implications

Given that participants were aware of their assigned interventions, the study is subject to potential performance and placebo bias, which may have influenced the recorded physiological responses. The absence of a sensory placebo in the control group may have further contributed to expectancy effects. Furthermore, limiting the intervention to the treatment phase may have reduced its efficacy, as pre‐procedural exposure—such as in the waiting area—could produce greater anxiolytic effects.

Variability in the outcomes of studies examining music and aromatherapy may be attributed to several methodological differences, including the underlying cause of anxiety, the type and invasiveness of the dental procedure, the age of the participants, the timing and setting of the intervention (e.g., waiting room vs. operatory), and inconsistencies in the type, dosage, and duration of aroma or music used. Future studies should standardize and report these parameters to enhance comparability and reproducibility.

Further research is warranted to assess the impact of music and aromatherapy applied before treatment (e.g., in the waiting room) and during shorter procedures, such as those lasting less than 1 h. Additional modalities, including virtual reality and pharmacologic interventions, should also be investigated as alternative or adjunctive strategies for anxiety management. Moreover, future studies are encouraged to incorporate validated psychological assessment tools—such as the State‐Trait Anxiety Inventory (STAI), the Beck Anxiety Inventory (BAI), or the Modified Dental Anxiety Scale (MDAS)—alongside physiological measurements. A combined approach may reveal new correlations and contribute to a more comprehensive understanding of dental anxiety in clinical contexts.

## 5 Conclusions

The present findings suggest that music and aromatherapy, when applied during endodontic treatment, do not significantly reduce blood pressure and heart rate. Accordingly, clinicians should consider other evidence‐based approaches to foster a more comfortable and anxiety‐reducing environment for their patients.

## Author Contributions

**Seyed Amir Mousavi:** conceptualization, funding acquisition, writing – review and editing. **Fateme Nasiri:** validation, data curation, writing – review and editing. **Melika Sadat Araghbidi Kashani:** investigation, writing – original draft preparation. **Ali Ghazalgoo:** project administration, supervision, writing – original draft preparation. **Pedram Iranmanesh:** methodology, resources, writing – review and editing. **Soheil Shahbazi:** formal analysis, writing – review and editing.

## Ethics Statement

The local ethics committee approved the study (IR.MUI.RESEARCH.REC.1398.526).

## Consent

Written consent forms were obtained from all participants.

## Conflicts of Interest

The authors declare no conflicts of interest.

## Supporting information

Supporting File 1 CONSORT Checklist.

Supporting File 2 FLOW.

## Data Availability

The data that support the findings of this study are available from the corresponding author upon reasonable request.
