# Supplementary material for: Effects of Music and Aromatherapy on Blood Pressure and Heart Rate Among Endodontic Patients: A Randomized Clinical Trial
Source: Clin Exp Dent Res. 2025 Jun 11;11(3):e70156. doi: 10.1002/cre2.70156 (PMC12152766; doi:10.1002/cre2.70156)
Supplement: Supplementary file 2 — Supporting File 2 FLOW. [file CRE2-11-e70156-s001.docx]

**Supplementary file S2**. The flowchart of the trials

Control group: receive neither music nor aromatherapy

Music

Aroma

Both

Divided into 4 groups

The endodontic treatment took approximately one hour to complete

In the treatment room, the systolic and diastolic blood pressure as well as the heart rate were recorded for each patient before administering anesthesia

No differences were found between the intervention groups and the control group

After the temporary filling material was placed, all parameters were recorded

The differences in data before and after the intervention were compared

Music group: received music and distilled water (as the placebo for aromatherapy)

Combined music and aromatherapy group: received music and lavender aroma

Aromatherapy group: received lavender aroma without music

Control

72 patients were referred to dental school
